# Iron functionalized silica particles as an ingenious sorbent for removal of fluoride from water

**DOI:** 10.1038/s41598-023-34357-8

**Published:** 2023-05-17

**Authors:** Paul Kiprono, Jackson Kiptoo, Eunice Nyawade, Elijah Ngumba

**Affiliations:** Department of Chemistry, School of Mathematics and Physical Sciences, Jomo Kenyatta University of Agriculture and Technology, P.O Box 62000-00200, Nairobi, Kenya

**Keywords:** Environmental sciences, Natural hazards, Chemistry, Materials science

## Abstract

The paucity of safe drinking water remains a global concern. Fluoride is a pollutant prevalent in groundwater that has adverse health effects. To resolve this concern, we devised a silica-based defluoridation sorbent from pumice rock obtained from the Paka volcano in Baringo County, Kenya. The alkaline leaching technique was used to extract silica particles from pumice rock, which were subsequently modified with iron to enhance their affinity for fluoride. To assess its efficacy, selected borehole water samples were used. Scanning electron microscopy, X-ray diffraction, Fourier transform infrared and X-ray fluorescence spectroscopy was used to characterize the sorbent. The extracted silica particles were 96.71% pure and amorphous, whereas the iron-functionalized silica particles contained 93.67% SiO_2_ and 2.93% Fe_2_O_3_. The optimal pH, sorbent dose and contact time for defluoridation of a 20 mg/L initial fluoride solution were 6, 1 g and 45 min, respectively. Defluoridation followed pseudo-second-order kinetics and fitted Freundlich's isotherm. Fluoride levels in borehole water decreased dramatically; Intex 4.57–1.13, Kadokoi 2.46–0.54 and Naudo 5.39–1.2 mg/L, indicating that the silica-based sorbent developed from low-cost, abundant and locally available pumice rock is efficient for defluoridation.

## Introduction

Groundwater is the most readily accessible source of drinking water, yet it is also the most polluted^1,2^. Fluoride is one of these pollutants, although at low levels it is also essential in the body as a trace element for the development of teeth and bones^3,4^. Prolonged exposure to high fluoride levels can cause dental and skeletal fluorosis, as well as harm to the kidneys, liver, brain and thyroid glands^5,6^. Over 260 million people worldwide are exposed to high fluoride levels through groundwater in the East Africa’s Rift Valley, Asia, Europe and America^7–9^. This has been attributed to geogenic processes such as volcanic activities and weathering of fluoride-rich minerals^10,11^. Fluoride enrichment in groundwater is also aided by effluents from the fertilizer, ceramic, pesticide, glass, aluminium and refrigerant industries^12–14^. Today, the World Health Organization (WHO) has established the allowable limit of fluoride in drinking water at 1.5 mg/L^15^, hence defluoridation processes such as ion exchange, adsorption, coagulation, precipitation and reverse osmosis are crucial to maintaining fluoride levels within this range^12,16^. However, the majority of these techniques are expensive to maintain and operate. Another constraint is the production of toxic sludge through methods such as precipitation, coagulation, and membrane filtration. Furthermore, techniques such as reverse osmosis and ion exchange are complicated and expensive, necessitating the use of water adsorbents^17,18^. Adsorption is the most preferred water purification technique because it is cheap, efficient, does not generate sludge, is simple to operate, and does not need electric power or specialized skills to operate. In addition, the adsorbents can be regenerated and reused making them the best at the household level and in small communities in less developed rural areas^19^. Commercial activated carbon derived from coal is among the most effective adsorbent for fluoride removal from water. It has a high specific surface area and is highly porous, however it is extremely expensive and has regeneration difficulties^17^. Other effective materials include bauxite^20^, bone char, metal oxides, polymer materials, biosorbents^21^, agricultural wastes^6^, sea materials, fly ash, carbonaceous materials^22^, nanoparticles^23^ and geomaterials^24^, all of which are low in cost and readily available, as is the case of silica mineral (SiO_2_). Silica is an auspicious materials with distinct features that satisfy almost all of the selection criteria for ideal water purification adsorbents, such as chemical inertness, structural and thermal stability, high specific surface area, non-toxicity, large pore size and the presence of surface functional groups (–Si–OH and –Si–O–Si–) that are readily modified to enhance selectivity towards a target pollutant^25^. Furthermore, it is abundant and widely distributed in nature, particularly in volcanic rocks such as pumice (60–70%)^26,27^. It is abundant in Kenya along the Rift Valley System in volcanic centers such as the Barrier, Namanuru, Emuruangogolak, Silali, Paka, Korosi, Menengai, Longonot, and Suswa craters^28^. Mourhly et al. demonstrated that it is feasible to isolate cost-effective silica particles from pumice volcanic rock using an alkaline extraction protocol at low temperatures. This method yielded 94% pure amorphous silica nanoparticles with a high specific surface area (422 m^2^g^−1^) and a mean pore diameter of 5.5 nm that was used as a support material for catalysis^29^. As previously stated, defluoridation has been accomplished using a variety of techniques and adsorbents. However, based on review of the literature, we are unaware of any reports of silica extracted from pumice rock and then modified with iron for fluoride removal from water. Therefore in this study, silica-based defluoridation sorbent was prepared by isolating silica particles from pumice rock via alkaline leaching then its surface modified with Fe^3+^ (hard acid) to increase selectivity towards F^−^ (hard base), and used to evaluate fluoride removal from water. Batch experiments were used to evaluate the kinetics and isotherm of fluoride adsorption, as well as the effects of pH, contact time, dosage and initial fluoride concentration on fluoride removal. The efficacy of the adsorbent was then assessed using borehole water samples.

## Materials and methods

### Study area and sample collection

With the assistance of a geologist, approximately 5 kg of pumice rock was collected at random in a clean well-label polythene sampling bag from Paka volcano in Baringo County, Kenya (36° 10′ 59″ E and 0° 55′ 14″ N).

### Chemicals and standards

The following analytical grade chemicals and reagents were used in this study: HCl, NaOH, H_2_SO_4_, NaF, pH buffers and total ionic strength adjustment buffer (TISAB) bought from Sigma-Aldrich through Kobian Scientific Limited in Kenya and used without further purification. Furthermore, deionized water was used throughout.

### Fluoride analysis

Fluoride levels were assessed using an ion-selective electrode (ISE) model (Elit 9801) in accordance with the American Public Health Association's standard protocol^30^.

### Pretreatment of pumice rock

Pumice rock samples were thoroughly cleaned with deionized water, dried and crushed. The ground powder was then passed through a 180 μm sieve to obtain uniform particle sizes, which were subsequently activated in a muffle furnace model (STT-1200C-3.5-12) at 500 °C for 3 h.

### Extraction of silica particles (SPs) from pumice rock

Silica particles were recovered in triplicate from pumice rock using a low-temperature alkaline leaching protocol described by Mourhly et al.^29^. In brief, 10 g of ground pumice was refluxed with 300 mL of 3 M NaOH at 100 °C for 4 h while stirring at 300 rpm to dissolve the silicate and form a Na_2_SiO_3_ solution^31^. To recover Na_2_SiO_3_, the slurry was filtered with ashless filter paper (Whatman No 41). The filtrate was then acidified with drops of 5 M H_2_SO_4_ to pH 7 while vigorously stirring to form silica gel^32^. Prior to filtration and thorough washing, the silica gel was aged overnight. The silica gel was then dried overnight at 110 °C before being refluxed with 1 M HCl for 3 h at 100 °C to remove any soluble minerals such as Fe, Al, Ca, and Mg. The suspension was filtered, thoroughly washed, and dried overnight at 110 °C. The final product was activated for 3 h in a muffle furnace at 550 °C to yield very fine white silica particles (SPs) powder.

#### Silica yield

The amount of silica recovered from from pumice rock was calculated using Eq. (1) ^33^.

1 $$SPs \; yield \left(\%\right)=\left(\frac{Average \; weight \; of \; exctracted \; SNPs \; (g)}{Average \; weight \; of \; silica \; in \; pumice \; rock \; (g)}\right)\times 100$$

The average weight of silica in pumice rock is the product of the average weight of pumice rock used in the extraction and the average percent SiO_2_ obtained from XRF analysis.

### Modification of SPs with iron

The silica particles were iron-coated according to the methodology established by Ref.^34^. In a 50 mL solution containing 1 g of Fe(NO_3_)_3_·9H_2_O, 10 g of silica particles were dissolved. The pH of the solution was adjusted to 7 with 0.5 M NaOH and then stirred at room temperature for 1 h. The mixture was centrifuged, and the resulting particles were thoroughly washed and dried overnight at 105 °C. Finally, the Fe-coated silica particles (FCSPs) were activated in a muffle furnace for 6 h at 500 °C before being stored in a clean plastic container.

### Characterization

The bulk chemical composition of pumice rock, silica particles (SPs) and Fe-coated silica particles (FCSPs) were determined using X-ray fluorescence (XRF) spectrophotometer (Rigaku ZSX Primus II). For phase identification, an X-Ray diffractometer (XRD) model (Rigaku MiniFlex II) with copper radiation (CuKα = 1.5418 Å) operating at 15 mA and 30 kV was used to record diffractograms between 2θ of 3° and 50°, with a step size of 0.02 at 2 s per step. The functional groups were identified using a Shimadzu fourier transform infrared (FTIR) spectrophotometer (IRAffinity-1S) in attenuated total reflectance mode, with spectra recorded between 4000 and 400 cm^−1^ with a resolution of 4 cm^−1^. The morphology of the silica particles was examined using a scanning electron microscope (JCM-7000-JEOL).

### Adsorption studies

A batch experiment was conducted at room temperature to determine the optimal pH, sorbent dose, contact time and initial fluoride concentration for fluoride removal using FCSPs. Equations (2) and (3) were used to calculate the amount of fluoride adsorbed at equilibrium (${\mathrm{q}}_{\mathrm{e}}$) and the percentage of fluoride removed^35^.

2 $${\mathrm{q}}_{\mathrm{e}}= \frac{\mathrm{V}({\mathrm{C}}_{\mathrm{o}}- {\mathrm{C}}_{\mathrm{e}} )}{\mathrm{M}}$$

3 $$\mathrm{\% \; Sorption}= \frac{{\mathrm{C}}_{\mathrm{o}}-{\mathrm{C}}_{\mathrm{e}}}{{\mathrm{C}}_{\mathrm{o}}} \times 100$$

where M (g) is the sorbent mass, V (L) is the volume of the solution, ${\mathrm{q}}_{\mathrm{e}}$(mg/g) is the amount of fluoride adsorbed at equilibrium, ${\mathrm{C}}_{\mathrm{o}}$ and ${\mathrm{C}}_{\mathrm{e}}$ (mg/L) is the initial and equilibrium fluoride concentrations, respectively^36^.

#### Optimization of pH

The effect of pH on fluoride removal was investigated using 1.5 g of FCSPs and 250 mL of a 20 mg/L fluoride solution. The pH was varied from 2 to 10 using 0.05 M HCl and 0.05 M NaOH. The solutions were stirred at room temperature for 90 min before being filtered with Whatman No. 42 filter paper. The residual fluoride concentration in the filtrate was then determined using an ion-selective electrode (ISE).

#### Optimization of sorbent dose

The effect of sorbent dose on defluoridation was evaluated by equilibrating various sorbent doses (0.2–2.5 g) with 250 mL of a 20 mg/L fluoride solution at the optimum pH of 6. The solutions were stirred at room temperature for 90 min before being filtered with Whatman No. 42 filter paper. The residual fluoride concentration in the filtrate was then determined using an ISE.

#### Optimization of contact time

The adsorption capacity of FCSPs as a function of time was studied using 250 mL of a 20 mg/L initial fluoride solution at optimal pH (6) and sorbent dose (1 g) by varying contact time from 5 to 90 min. After stirring the solutions for a predetermined time at room temperature, they were left to settle for 2 min before being filtered with Whatman No. 42 filter paper. The concentration of residual fluoride in the filtrates was then determined using an ISE.

#### Optimization of initial fluoride concentration

The effect of initial fluoride concentration on defluoridation was investigated using optimal pH (6), dose (1 g) and contact time (45 min), and the initial fluoride concentration was varied from 2 to 60 mg/L. After stirring the solutions for 45 min at room temperature, they were left to settle for 2 min before being filtered with Whatman No. 42 filter paper. The concentration of residual fluoride in the filtrates was then determined using an ISE.

#### Adsorption isotherms

In this study, the Langmuir and Freundlich models were used to interpret adsorption data^37^. Freundlich model usually describes a heterogeneous system based on assumption that sorption takes place in several sites and as the number of adsorbates increases, the surface binding energy decreases exponentially which implies a multilayer formation. The model is expressed by Eqs. (4) and (5) ^38^.

4 $${\mathrm{q}}_{\mathrm{e}}={\mathrm{K}}_{{\mathrm{F}}}{{\mathrm{C}}}_{{\mathrm{e}}}^{1/n} \left({\text{Non-linear form}}\right)$$

5 $${\mathrm{Log q}}_{\mathrm{e}}= {\mathrm{Log K}}_{\mathrm{F}}+ \frac{1}{\mathrm{n}} {\mathrm{Log C}}_{\mathrm{e}} \left(\mathrm{Linear \; form}\right)$$

where ${\mathrm{C}}_{\mathrm{e}}$ (mg/L) is the concentration of fluoride at equilibrium. ${\mathrm{q}}_{\mathrm{e}}$ (mg/g) is the amount of fluoride adsorbed per unit mass of adsorbent. ${\mathrm{K}}_{\mathrm{F}}$ (mg/g) is the Freundlich coefficient indicating sorbent sorption capacity. 1/n (unitless) is the constant, signifying surface heterogeneity or adsorption intensity with a value ranging from 0.1 to 1^39^. The Langmuir model essentially describes a monolayer type of adsorption and it is expressed by Eq. (6)^40^.

6 $$\frac{{\mathrm{C}}_{\mathrm{e}}}{{\mathrm{q}}_{\mathrm{e}}}= \frac{{\mathrm{C}}_{\mathrm{e}}}{{\mathrm{q}}_{\mathrm{max}}}+\frac{1}{{\mathrm{K}}_{\mathrm{L }}\times {\mathrm{q}}_{\mathrm{max}}}$$

where ${\mathrm{q}}_{\mathrm{e}}$ (mg/g) is the amount of fluoride adsorbed per unit mass of adsorbent. ${\mathrm{C}}_{\mathrm{e}}$ (mg/L) is the concentration of fluoride at equilibrium. ${\mathrm{q}}_{\mathrm{max}}$ (mg/g) is the maximum monolayer adsorption capacity. ${\mathrm{K}}_{\mathrm{L}}$ is the Langmuir constant depicting adsorbent affinity towards the adsorbate.

The value of the separation factor (R_L_) expressed by Eq. (7) indicates the suitability of the Langmuir model to fit the data:

7 $${R}_{L}=\frac{1}{1+{K}_{L}{C}_{o}}$$

The value of R_L_ indicates whether the isotherm is favourable (0 < R_L_ < 1), unfavourable (R_L_ > 1), linear (R_L_ = 1) or irrevesible (R_L_ = 0).

#### Kinetics models

Pseudo-first-order and pseudo-second-order kinetics models were used to investigate the rate and mechanism of defluoridation^39^. Pseudo-first-order is ideal for simple sorption processes in which saturation occurs in 20–30 min^41^ and it is expressed by Eq. (8) ^42,43^.

8 $$\frac{d{q}_{t}}{dt}= {K}_{1 }\left({q}_{e }-{q}_{t}\right)$$

Integrating and linearizing Eq. (8) yields Eqs. (9) or (10)^44^.

9 $$\mathrm{log }({q}_{e }-{q}_{t})=\mathrm{log}{q}_{e}- \frac{{K}_{1}}{2.303} t$$

10 $$\mathrm{ln}({q}_{e }-{q}_{t})= \mathrm{I}n{q}_{e}- {K}_{1}t$$

where ${\mathrm{q}}_{\mathrm{t}}$ and ${\mathrm{q}}_{\mathrm{e}}$ are fluoride concentrations (mg/g) at a time (t) and equilibrium, respectively, and ${\mathrm{K}}_{1}$ (min^−1^) denotes the rate constant. Plotting $\mathrm{log }\left({q}_{e }-{q}_{t}\right)$ versus time yields a straight line and the values for ${\mathrm{q}}_{\mathrm{e}}$ and ${\mathrm{K}}_{1}$ are determined from the intercept and slope, respectively^43^.

### Removal of fluoride from real water samples

Borehole water samples collected from Tiaty in Baringo County, Kenya, were utilized to evaluate the efficiency of FCSPs in defluoridation. Apart from filtration with Whatman No. 42 filter paper, the samples were used without any other treatments. The initial fluoride levels were determined, then defluoridation was performed using the optimal sorbent dose (1 g) and contact time (45 min). The residual fluoride levels were then determined.

### Regeneration studies

A batch desorption experiment was done according to Rafigue and colleagues with slight modification to evaluate the ability of adsorbents to be regenerated and recycled^13^. Five consecutive cycles of adsorption–desorption experiments were done using 0.1 M NaOH as a desorbing agent. The spent sorbent was soaked in NaOH for 2 h, washed with deionized water until the washed water pH was 7 then dried in an oven at 90 °C for 4 h. A fluoride solution of 20 mg/L initial concentration was used with optimum sorbent dose (1 g) and contact time (45 min).

## Results and discussion

### Silica yield

From an average of 9.978 g of pumice rock used, 5.296 g silica particles (SPs) were recovered. According to Eq. (1). This implies that silica particle extraction from pumice rock via alkaline leaching is viable. Previous research has revealed a similar outcome^29^.

### Characterization

#### XRF analysis

Table 1 shows the chemical components of pumice rock, silica particles (SPs), and Fe-coated silica particles (FCSPs) derived from XRF analysis. The main components are SiO_2_ (61.41%), Al_2_O_3_ (12.07%) and Fe_2_O_3_ (11.06%).

**Table 1:** Chemical composition of pumice rock, SPs, and FCSPs.

Components	Composition (% w/w)		
	Pumice rock	SPs	FCSPs
SiO_2_	61.41	97.71	93.67
Al_2_O_3_	12.07	–	–
Fe_2_O_3_	11.06	–	2.93
CaO	1.11	–	–
MgO	0.18	–	–
SO_3_	0.1	–	–
K_2_O	5.51	–	–
Na_2_O	6.36	–	–
P_2_O_5_	0.08	–	–
MnO	0.45	–	–
Loss on ignition	1.67	2.29	3.4

Similarly, in previous research, SiO_2_ was reported to be the most abundant component of pumice rock, accounting for 61.6%^27^ and 63.4%^26^. As demonstrated in Table 1, the isolated SPs contained exclusively SiO_2_. The absence of other oxides previously present in raw pumice rock, along with the high silica content of 96.71%, imply that relatively pure SPs were extracted. SiO_2_ and Fe_2_O_3_ contents in FCSPs were 93.67% and 2.93%, respectively. The reduction in SiO_2_ from 96.71% (SPs) to 93.67% (FCSPs) with the addition of Fe_2_O_3_, which wasn't present in pure SPs, reveals that the iron coating of SPs was effective.

#### XRD analysis

**Figure 1:**
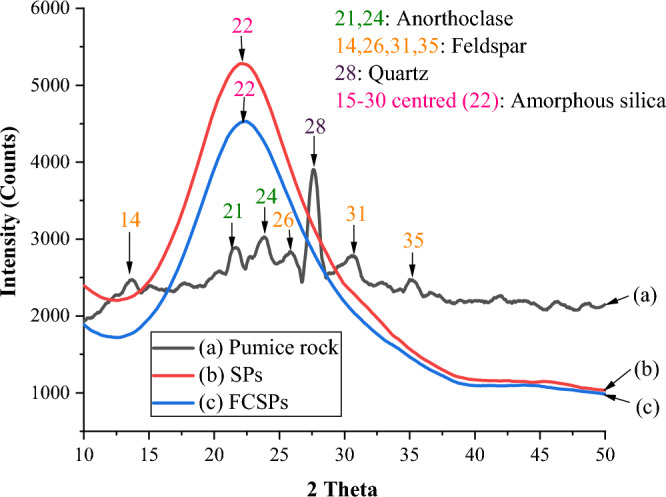
X-ray diffractograms of (**a**) pumice rock, (**b**) silica particles, and (**c**) Fe-coated silica particles.

An X-ray diffractometer was used to identify the minerals present in pumice rock, SPs, and FCSPs. According to the diffractograms in Fig. 1, pumice rock comprises crystalline phase minerals, primarily anorthoclase, feldspar and quartz^45^.

The extracted silica particles exhibited a single broad peak from 2θ of 15° to 30°, centered at 2θ of 22°, which is a distinctive feature of amorphous silica^46^. The absence of crystalline peaks previously observed in pumice rock confirms that the isolated SPs were predominantly amorphous^31^. The Fe-coated silica particles were likewise amorphous.

#### FTIR analysis

**Figure 2:**
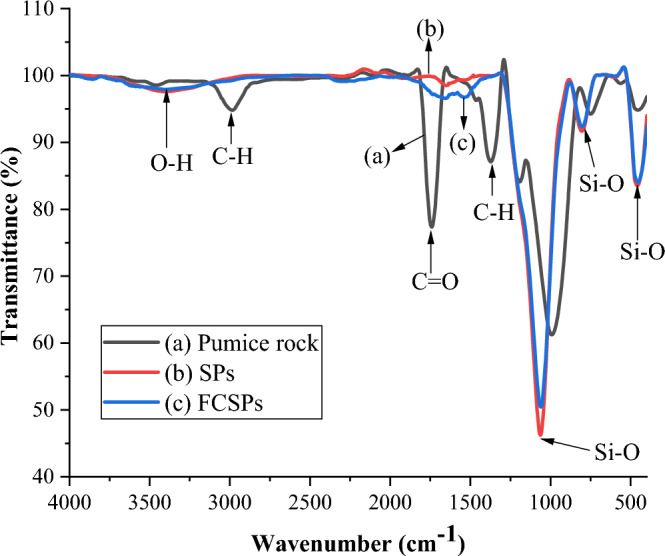
FTIR spectrum of pumice rock (**a**), silica particles (**b**) and Fe-coated silica particles (**c**).

The functional groups present in pumice rock, SPs and FCSPs are depicted in Fig. 2.

The stretching vibration of the O–H bond from the silanol group (Si–OH) is responsible for the broad peak detected between 3000 and 3700 cm^−1^ and centered at 3352 cm^−1^^32,33^. A strong band at 1048 cm^−1^ corresponds to asymmetric stretching of the Si–O bond, whereas bands at 454 and 789 cm^−1^ relate to bending and asymmetric vibrations of the Si–O bond in the siloxane group, respectively^47^. The bands at 2985, 1741 and 1375 cm^−1^ on the pumice rock are attributed to the C–H stretch, C=O stretch and C–H bend, respectively^48^.

#### SEM analysis

**Figure 3:**
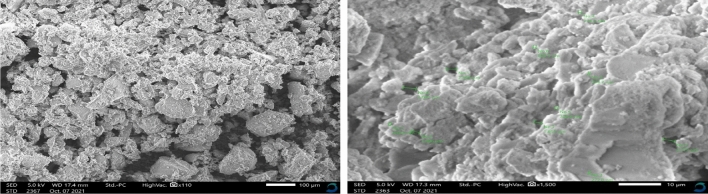
SEM micrographs for silica particles at different magnifications (left × 110 and right × 1500).

The SEM micrographs in Fig. 3 demonstrate that the extracted silica particles were spherical and agglomerated together to form clusters. This denotes amorphous silica and is consistent with XRD data (Fig. 1). A similar finding was made when silica particles were extracted from pumice rock^29^.

### Adsorption studies

#### Effect of pH

**Figure 4:**
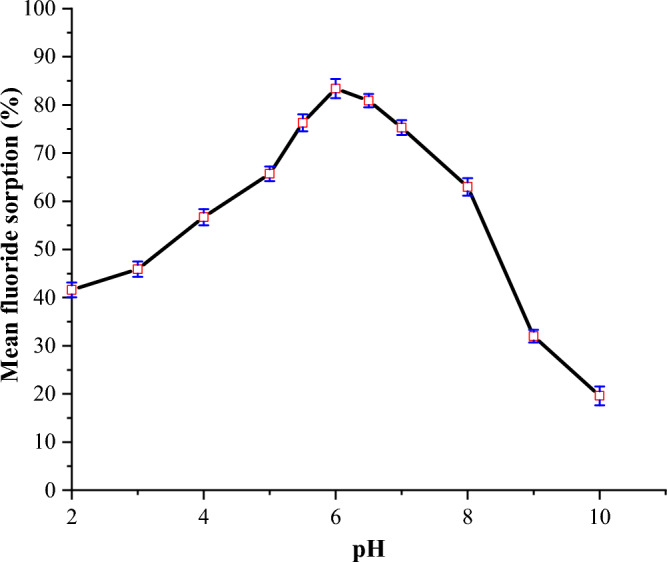
Effect of pH on the adsorption capacity of FCSPs (initial fluoride concentration = 20 mg/L, sorbent dose = 1.5 g and contact time = 90 min).

The effect of pH on the removal of fluoride from water by FCSPs was investigated, and the results are shown in Fig. 4.

As illustrated in Fig. 4, fluoride sorption rose from 41.6% at pH 2 to an optimum of 83.4% at pH 6, and then decreased as pH increased further. The pH of the solution is an important parameter in the adsorption process since it regulates the sorbent's surface charge and the degree of ionization of the adsorbate^49^. The reduced sorption capacity at low pH could be due to the generation of weakly ionizing hydrofluoric acid, which decreases the availability of free fluoride ions for electrostatic interactions with Fe^3+^ on the sorbent surface^7,49^. The declines in sorption capacity from 83.4 to 19.6% with pH rises from 6 to 10 may be attributed to competition for the active site on the adsorbent between OH^-^ and F^-^ ions due to their similar ionic sizes and charges^24^. Furthermore, the decrease in sorption capacity at alkaline pH can be due to the electrostatic repulsion of fluoride ions with the negatively charged adsorbent surface^9^.

#### Effect of sorbent dose

**Figure 5:**
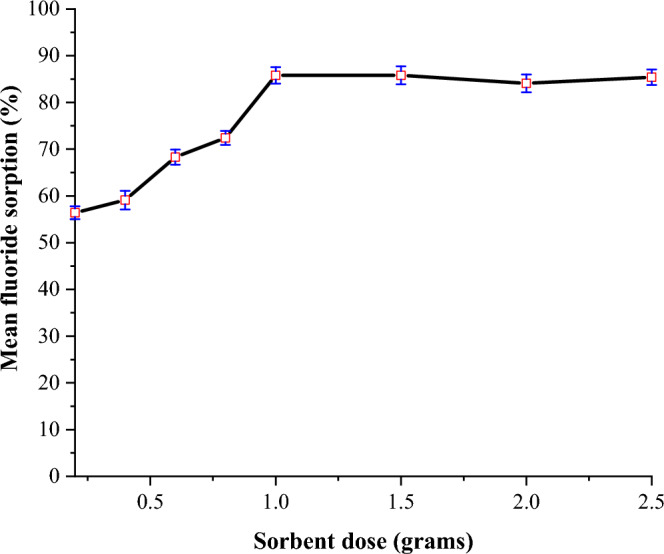
Effect of sorbent dose on the adsorption capacity of FCSPs (initial fluoride concentration = 20 mg/L, pH = 6 and contact time = 90 min).

The effect of sorbent dose on defluoridation was investigated by varying the sorbent dosage from 0.2 to 2.5 g at the optimal pH of 6. Figure 5 depicts the outcomes.

The results show that increasing the sorbent dose from 0.2 to 1.0 g increases fluoride removal from 56.4 to 85.8%. According to Nehra and co-workers, this is most likely owing to the availability of a greater number of unoccupied active sorption sites and the existence of more surface areas for sorption^50^. However, increasing the sorbent dose from 1.0 to 2.5 g has no discernible effect on sorption capacity, presumably due to sorbent agglomeration or overlap, which reduces the availability of active sorption sites at higher sorbent doses^51^. In earlier studies, most adsorbents showed a similar trend^14,52^.

#### Effect of contact time

**Figure 6:**
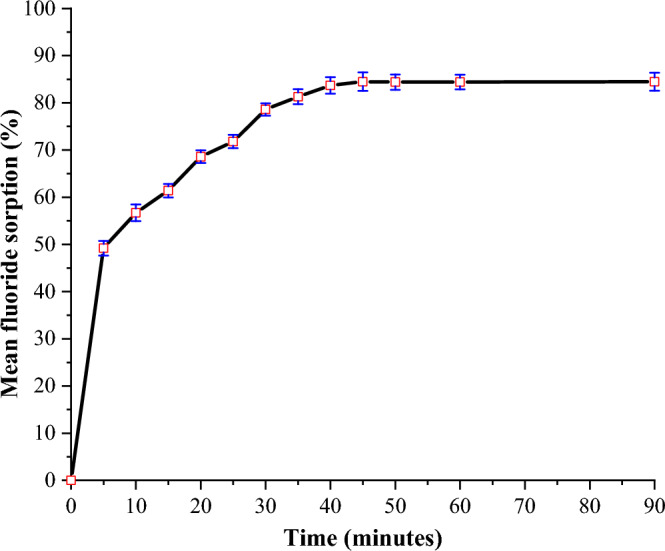
Effect of contact time on the adsorption capacity of FCSPs (initial fluoride concentration = 20 mg/L, pH = 6 and sorbent dose = 1 g).

The effect of contact time on fluoride removal was studied by varying contact time from 5 to 90 min using optimum pH (6) and sorbent dose (1 g). Figure 6 depicts the results.

Fluoride sorption increased rapidly in the beginning, from 49.2 to 84.5% at 5 and 45 min (Fig. 6). The presence of a higher number of vacant active sites and a fluoride concentration gradient may be responsible for the initial high fluoride sorption rate^49^. After 45 min, there were negligible changes in fluoride uptake, presumably due to a decrease in the number of active sites and fluoride concentration^14^.

#### Effect of initial fluoride concentration

**Figure 7:**
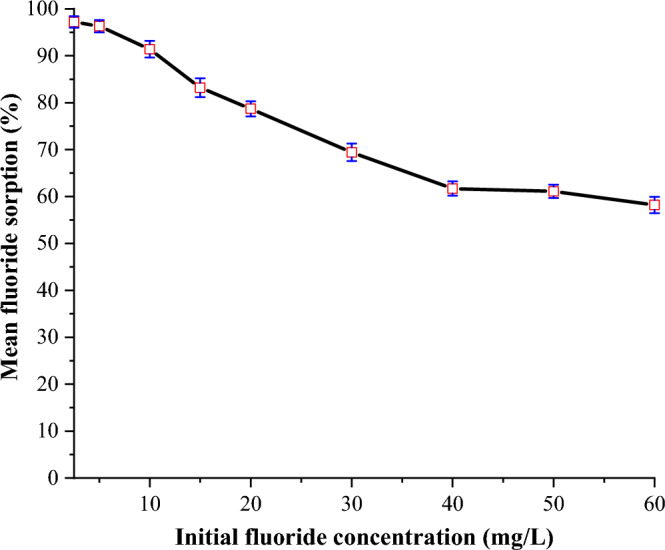
Effect of initial fluoride concentration on the sorption capacity of FCSPs (initial fluoride concentration = 20 mg/L, pH = 6 and contact time = 45 min).

The effect of initial fluoride concentration on fluoride removal was investigated at room temperature by varying the initial fluoride concentration from 2 to 60 mg/L while utilizing the optimum pH (6), sorbent dose (1 g) and contact time (45 min). Figure 7 depicts the outcomes.

Fluoride absorption is greater when the initial fluoride concentration is lower than when the initial fluoride concentration is higher (Fig. 7). This means that the sorbent's capability diminishes as the initial fluoride concentrations rise. This could be ascribed to sorbent active site saturation as a result of a larger fluoride-to-sorbent active site ratio^53^. Previous research has also shown that as the initial fluoride concentration increases, the sorbent's fluoride removal ability diminishes^41,54,55^.

### Adsorption isotherms

**Figure 8:**
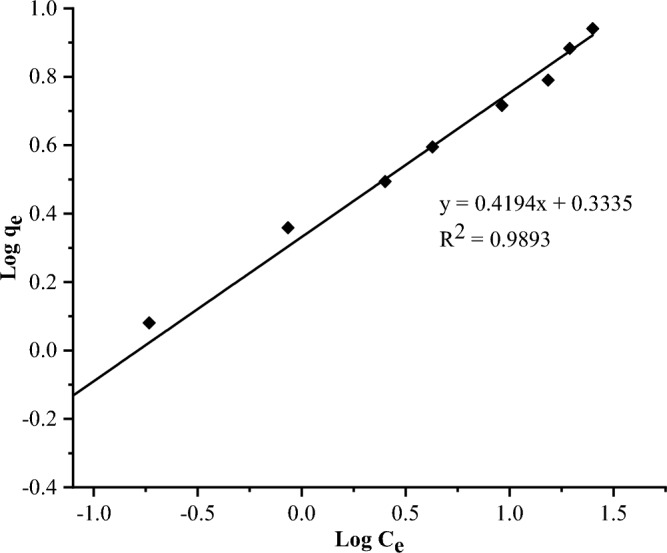
Freundlich adsorption isotherm plot.

**Figure 9:**
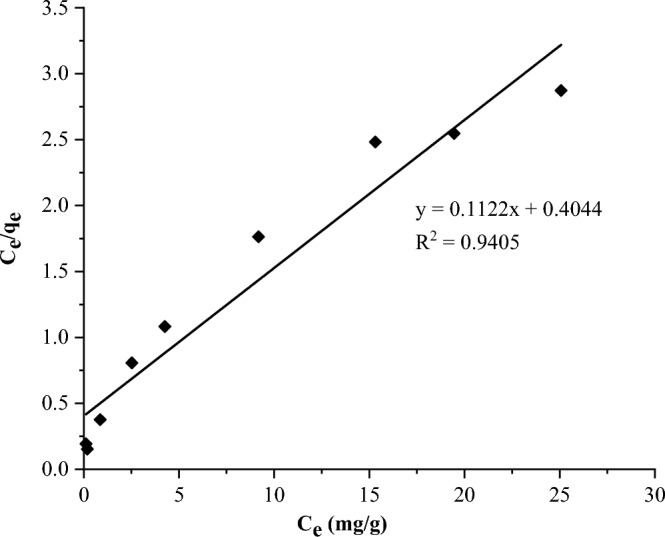
Langmuir adsorption isotherm plot.

The Freundlich and Langmuir models were used to interpret the data from adsorption experiment. The plots are presented in Figs. 8 and 9, respectively.

Table 2 shows that the experimental data fit better to the Freundlich isotherm model (R^2^ = 0.989) than the Langmuir isotherm (R^2^ = 0.941). The values of 1/n (0.419) between 0.1 and 1.0 and n (2.384) between 1 and 10 confirmed the high bond strength between the adsorbate and adsorbent, as well as the heterogeneous nature of the adsorbent surface. Furthermore, the low value of 1/n indicates the heterogeneity of the adsorbent surface^13^. The small value of the Langmuir constant (K_L_), 0.277 L/mg, implies a low heat of adsorption^56^. The R_L_ value of 0.15 (Table 2), which is between 0 and 1, indicates favorable experimental conditions for sorption. According to the Langmuir model, q_max_ is 8.913 mg/g (Table 2).

**Table 2:** Calculated Freundlich and Langmuir isotherm parameters.

Freundlich isotherm					Langmuir isotherm					
Intercept	Slope (1/n)	n	K_F_	R^2^	Intercept	Slope	q_max_ (mg/g)	R_L_	K_L_ (L/mg)	R^2^
0.334	0.419	2.384	2.155	0.989	0.404	0.112	8.913	0.150	0.277	0.941

### Kinetics of defluoridation

**Figure 10:**
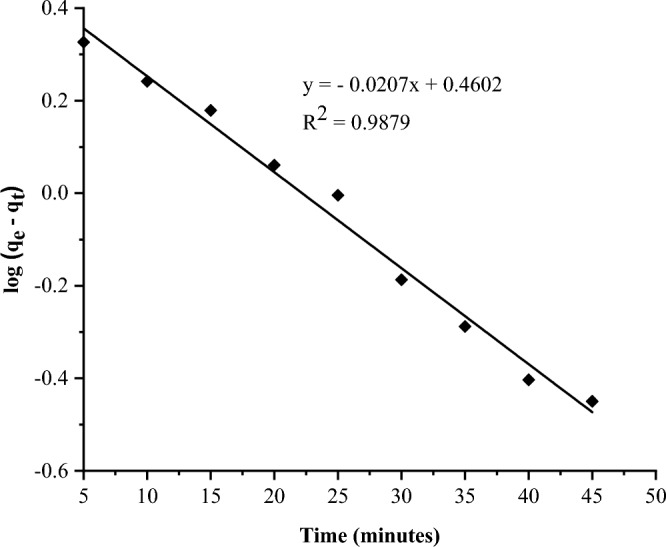
Pseudo-first-order kinetics plot.

**Figure 11:**
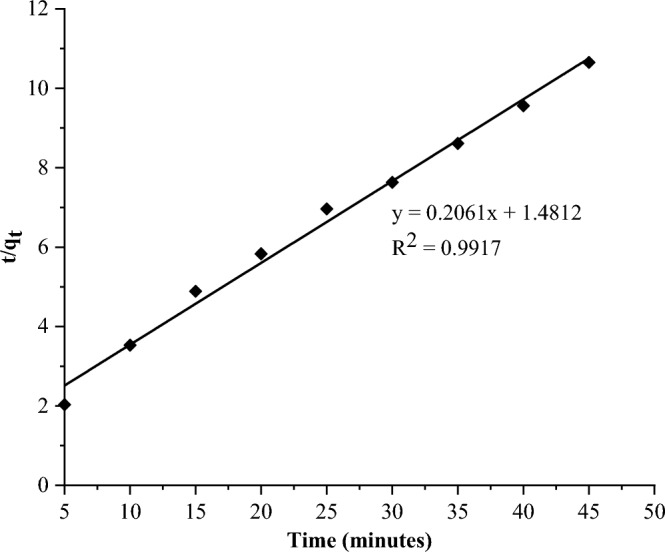
Pseudo-second-order kinetics plot.

The rate as well as mechanism of defluoridation was evaluated using pseudo-first-order and pseudo-second-order kinetics models. The plots are presented in Figs. 10 and 11, respectively.

The linear regression plots show that the experimental data fit best to the pseudo-second-order model, which has a higher correlation coefficient of R^2^ = 0.992 (Table 3), than the pseudo-first-order model (R^2^ = 0.988).

**Table 3:** Kinetics models constants.

Pseudo-first-order					Pseudo-second-order				
Slope	K_1_	Intercept	q_e_	R^2^	Slope	q_e_	Intercept	K_2_	R^2^
− 0.021	0.048	0.460	2.885	0.988	0.206	4.852	1.481	0.029	0.992

The fit of this data to a pseudo-second-order model shows that adsorption occurs via chemisorption caused by electrostatic attractions or, more likely, ion exchange processes^54,57^. These findings are consistent with the majority of previous studies on fluoride removal using various adsorbents, as shown in Table 4.

**Table 4:** Comparison of adsorption capacity of FCSPs with different adsorbents.

Adsorbent	pH	Fitted kinetic model	Isotherm model	Adsorption capacity (mg/g)	References
Diatomite modified with aluminium hydroxide	6.7	Pseudo-second-order	Freundlich	1.67	^54^
Nano silica from rice husk	8	Pseudo-second-order	Freundlich	12	^55^
Aluminium hydroxide-loaded zeolite from coal fly ash	6	Pseudo-second-order	Langmuir	18.12	^58^
Fired clay pots	8	Pseudo-second-order	Freundlich	1.6	^56^
Natural clay (Kaolinite)		Pseudo-second-order	Freundlich	3.74	^59^
Marble waste powder	7	Pseudo-second-order	Freundlich	1.2	^60^
FCSPs	6	Pseudo-second-order	Freundlich	8.913	Current study

### Application of FCSPs to real water samples

**Figure 12:**
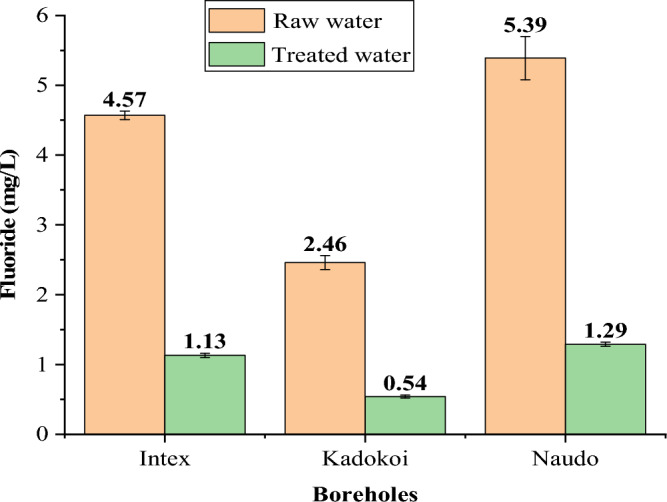
Comparison of fluoride levels in raw and treated groundwater.

Water samples collected from Tiaty Baringo County in Kenya were utilized to examine the efficacy of FCSPs in defluoridation; the findings are displayed in Fig. 12.

The FCSPs adsorb a reasonable amount of fluoride from water, up to the WHO criterion of 1.5 mg/L^61^. However, the percent fluoride removal was lower than what could be obtained using the model solution, which is ascribed to competition for the sorbent active sites with other potential anions commonly found in groundwater such as PO_4_^3−^, Cl^−^, SO_4_^2−^ and NO_3_^−^_._

### Regeneration studies

**Figure 13:**
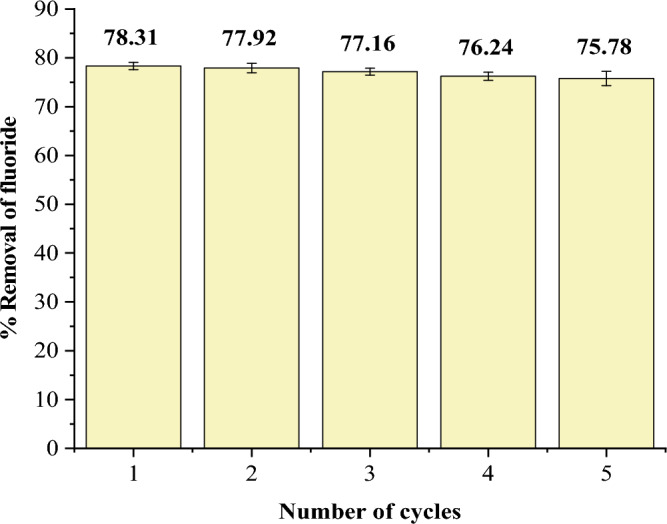
Regeneration of iron-functionalized silica particles.

Five adsorption–desorption cycles were performed to assess the adsorbent's ability to be regenerated and reused. The adsorption efficiency decreased with the number of cycles, but not significantly (Fig. 13). This implies that the adsorbent can be recycled several times without losing its efficiency, which is an important factor to consider when choosing an adsorbent.

## Conclusions

In this work, amorphous silica particles were isolated from pumice rock, coated with iron, and then utilized to assess fluoride removal in water. The primary components of pumice rock were SiO_2_ (61.41%), Al_2_O_3_ (12.07%), and Fe_2_O_3_ (11.06%). The extracted silica particles (SPs) were 96.71% pure and amorphous whereas the iron-coated silica particles (FCSPs) contained 93.67% SiO_2_ and 2.93% Fe_2_O_3_. The optimal pH, sorbent dose, and contact time for defluoridation 20 mg/L initial fluoride solution were 6, 1 g and 45 min, respectively. Fluoride absorption fit Freundlich's isotherm model, indicating multilayer fluoride absorption on a heterogeneous surface, whereas defluoridation followed pseudo-second-order kinetics, implying chemisorption. Fluoride levels in borehole water decreased dramatically; Intex 4.57 to 1.13, Kadokoi 2.46 to 0.54, and Naudo 5.39 to 1.2 mg/L. Furthermore, regeneration studies demonstrated that FCSPs can be recycled up to five times without losing efficiency significantly. As a result, the silica-based sorbent developed from readily available pumice rock is appropriate for removing fluoride from water. It is recommended that more research be done on the effects of competing anions such as PO_4_^3−^, Cl^−^, SO_4_^2−^ and NO_3_^−^ on the efficiency of fluoride removal using FCSPs.

## Data Availability

The data that support the findings of this work are accessible upon request from the corresponding author.
